# CASE REPORT REVIEW HIGHLIGHTING AN OUTPATIENT APPROACH IN ATYPICAL DIABETIC PRESENTATION IN YOUNG ADULT

**DOI:** 10.51894/001c.123045

**Published:** 2024-08-30

**Authors:** Marie Kenny

**Affiliations:** 1 Family Medicine Henry Ford Macomb

## 64

### INTRODUCTION

This case study is about the diagnosis and management of latent autoimmune diabetes in adults (LADA) that presents atypically in the outpatient setting. LADA affects 2-12% of all patients with adult-onset diabetes. Keeping LADA on the differential in an atypical presentation of elevated glucose has a significant impact on patient care and their outcomes. The purpose of this case study is to familiarize outpatient clinicians with the diagnosis and management of LADA to preserve pancreatic beta cell function. While there are key diagnostic criteria for LADA, it is important to note that these criteria are not categorical as LADA is functionally a hybrid of Type 1 and Type 2 diabetes.

### CASE DESCRIPTION

This study reviews the case of a 27-year-old male with no significant medical history who had presented to the emergency department with a three-month history of nausea and vomiting. He had a daily marijuana smoking habit, was a current smoker with a 15 pack-year history and consumed 5-6 soda pops daily. He was incidentally found to have an elevated glucose of 162 and was initially diagnosed with Type 2 diabetes. He was advised to establish care with a PCP. He presented to our residency clinic. An A1c was ordered and resulted at 7.3. However, due to his younger age and BMI of 22.38, an autoimmune work-up was also completed. His GAD antibodies were significantly elevated. This led to a diagnosis of latent autoimmune diabetes in adults (LADA), which requires different medical management compared to Type 2 diabetes. He was started on a DPP4 inhibitor and a non-dihydropyridine calcium channel blocker (CCB). Within three months of beginning this regimen, there was a significant drop in A1c from 7.3 to 5.2.

### CONCLUSION/LEARNING POINTS

This case highlights the importance of considering autoimmune causes for diabetes in younger adults, even in patients who report an unhealthy diet and lifestyle. It also emphasizes the harmful bias of quickly assigning a Type 2 diabetes diagnosis as it can delay appropriate interventions and negatively affect optimal outcomes for the patient.

